# Epidemiological Characteristics of OXA-232-Producing Carbapenem-Resistant Klebsiella pneumoniae Strains Isolated during Nosocomial Clonal Spread Associated with Environmental Colonization

**DOI:** 10.1128/spectrum.02572-21

**Published:** 2022-06-22

**Authors:** Xinhong Han, Ying Chen, Junxin Zhou, Qiucheng Shi, Yan Jiang, Xueqing Wu, Jingjing Quan, Huangdu Hu, Qian Wang, Yunsong Yu, Ying Fu

**Affiliations:** a Department of Infectious Diseases, Sir Run Run Shaw Hospital, Zhejiang University School of Medicine, Hangzhou, Zhejiang Province, China; b Key Laboratory of Microbial Technology and Bioinformatics of Zhejiang Province, Hangzhou, Zhejiang Province, China; c Regional Medical Center for National Institute of Respiratory Diseases, Sir Run Run Shaw Hospital, Zhejiang University School of Medicine, Hangzhou, Zhejiang Province, China; d Department of Clinical Laboratory, The Children’s Hospital, National Clinical Research Center for Child Health, Zhejiang University School of Medicine, Hangzhou, Zhejiang Province, China; e Department of Clinical Laboratory, Sir Run Run Shaw Hospital, Zhejiang University School of Medicine, Hangzhou, Zhejiang Province, China; f Key Laboratory of Precision Medicine in Diagnosis and Monitoring Research of Zhejiang Province, Hangzhou, Zhejiang Province, China; State Key Laboratory of Microbial Resources, Institute of Microbiology, Chinese Academy of Sciences

**Keywords:** *Klebsiella pneumoniae*, nosocomial clonal spread, OXA-232, transmission, environment colonization

## Abstract

Here, a program was designed to surveil the colonization and associated infection of OXA-232-producing carbapenem-resistant Klebsiella pneumoniae (CRKP) (OXA-232-CRKP) in an intensive care unit (ICU) and to describe the epidemiological characteristics during surveillance. Samples were sourced from patient and environment colonization sites in the ICU from August to December 2019. During the surveillance, 106 OXA-232-CRKP strains were isolated from 8,656 samples of colonization sites, with an average positive rate of 1.22%. The rate from patient colonization sites was 3.59% (60/1,672 samples), over 5 times higher than that of the environment (0.66% [46/6,984 samples]). Rectal swabs and ventilator-related sites had the highest positive rates among patient and environment colonization sites, respectively. Six of the 15 patients who had OXA-232-CRKP at colonization sites suffered from OXA-232-CRKP-related infections. Patients could obtain OXA-232-CRKP from the environment, while long-term patient colonization was mostly accompanied by environmental colonization with subsequent infection. Antimicrobial susceptibility testing presented similar resistance profiles, in which all isolates were resistant to ertapenem but showed different levels of resistance to meropenem and imipenem. Whole-genome sequencing and single-nucleotide polymorphism (SNP) analysis suggested that all OXA-232-CRKP isolates belonged to the sequence type 15 (ST15) clone and were divided into two clades with 0 to 45 SNPs, sharing similar resistance genes, virulence genes, and plasmid types, indicating that the wide dissemination of OXA-232-CRKP between the environment and patients was due to clonal spread. The strains all contained β-lactam resistance genes, including *bla*_OXA-232_, *bla*_CTX-M-15_, and *bla*_SHV-106_, and 75.21% additionally carried *bla*_TEM-1_. In brief, wide ST15 clonal spread and long-term colonization of OXA-232-CRKP between patients and the environment were observed, with microevolution and subsequent infection.

**IMPORTANCE** OXA-232 is a variant of OXA-48 carbapenemase, which has been increasingly reported in nosocomial outbreaks in ICUs. However, the OXA-232-CRKP transmission relationship between the environment and patients in ICUs was still not clear. Our study demonstrated the long-term colonization of OXA-232-CRKP in the ICU environment, declared that the colonization was a potential risk to ICU patients, and revealed the possible threat that this OXA-232-CRKP clone would bring to public health. The wide dissemination of OXA-232-CRKP between the environment and patients was due to ST15 clonal spread, which presented a multidrug-resistant profile and carried disinfectant resistance genes and virulence clusters, posing a challenge to infection control. The study provided a basis for environmental disinfection, including revealing common environmental colonization sites of OXA-232-CRKP and suggesting appropriate usage of disinfectants to prevent the development of disinfectant resistance.

## INTRODUCTION

Carbapenem-resistant Klebsiella pneumoniae (CRKP) infections are a major health problem worldwide (1). It was reported that 31% of patients who were colonized by carbapenemase-producing CRKP progressed to infection, and the rate was higher than that for patients colonized by non-carbapenemase-producing CRKP (9%) (2). The mechanism of carbapenem resistance in K. pneumoniae mainly relies on the production of carbapenemase, which can be characterized into different enzyme types, such as KPC, NDM, VIM, IMP, and OXA-48-like (3–5). Among them, OXA-48-like carbapenemases belong to the class D β-lactamases, which are endemic among *Enterobacterales* in the Middle East, North Africa, and European countries such as Belgium and Spain (6, 7). However, an increased prevalence of this enzyme type was recently observed in areas of nonendemicity, such as China. According to clinical research data, the proportion of OXA-48-like carbapenemases in *Enterobacteriaceae* isolated from patients in China increased from 0.1% in 2012 to 7.3% in 2019 (3, 8).

One of the OXA-48-like carbapenemases, OXA-232, has 5 amino acid differences in the β5-β6 loop, compared to OXA-48 (9), and the encoding gene *bla*_OXA-232_ is usually located on a 6,141-bp ColE-type nonconjugative plasmid. Compared to other OXA-48-like carbapenemases, OXA-232 has weaker carbapenemase activity (10). However, when combined with extended-spectrum β-lactamases or outer membrane protein mutations, the OXA-232 enzyme can cause high-level carbapenem resistance (11). The OXA-232 enzyme was first reported in Escherichia coli and K. pneumoniae strains from patients in France in 2013 and is currently endemic in India (12, 13). Recently, nosocomial infection outbreaks among patients with OXA-232-producing CRKP (OXA-232-CRKP) have also been reported worldwide, including pneumonia or urinary tract infections among neonatal or elderly patients in intensive care units (ICUs), while few reports have focused on the relationship between OXA-232-CRKP colonization and infection (11, 13–15). In addition, the ICU environment is a reservoir for outbreaks of multidrug-resistant pathogens, while the transmission of OXA-232-CRKP between the environment and patients has rarely been reported (16, 17). It is valuable to be aware of the epidemiological characteristics of OXA-232-CRKP in patients and in the environment for better prevention of CRKP dissemination.

In 2018, one OXA-232-CRKP strain was isolated from the ICU of Sir Run Run Shaw Hospital (SRRSH) in Hangzhou, China (18). To investigate whether OXA-232-CRKP is prevalent in patients and environmental settings, a surveillance program was performed in the same ICU in 2019. In this study, OXA-232-CRKP isolates were collected from patients and environmental colonization samples in the ICU of a teaching hospital in China. We aimed to surveil the colonization of OXA-232-CRKP in the environment and in patients in the ICU, to clarify the epidemiological characteristics, and to provide insights into the prevention and control of nosocomial infections caused by high-risk OXA-232-CRKP clones.

## RESULTS AND DISCUSSION

Previous reports have confirmed that Klebsiella pneumoniae is capable of colonization at various places within hospitals, such as sewage and water, and even in patients’ gastrointestinal tracts and nasopharyngeal tracts, which is an independent risk factor associated with nosocomial infection, especially in patients who are in an immunodeficient state (19, 20). Qin et al. reported that the infection incidence of patients who had CRKP colonization in the intestinal tract and nasopharyngeal tract was almost 45.9% in the ICU at Huashan Hospital (Shanghai, China), which posed a true threat to public health because antimicrobial agents are rarely used to treat multidrug- resistant pathogens (21). Although the environment has been considered a reservoir for pathogens in the ICU and nosocomial outbreaks of OXA-232-CRKP have increased in recent years, there is limited literature reporting on the relationship and transmission of OXA-232-CRKP between the environment and patients (9, 22, 23). In this study, we focused on the OXA-232-CRKP that was first reported by Potron et al. in 2013 and then spread throughout the world in recent years (12), whereas limited literature focuses on colonization in hospitals and patient infections.

Here, we designed a surveillance program in our 28-bed ICU to proactively monitor the colonization of OXA-232-CRKP, which presented multidrug resistance, and investigated the relationship between colonization and subsequent infection revealed by whole-genome sequencing (WGS). The results of our research affirmed the long-term colonization of OXA-232-CRKP sequence type 15 (ST15) in the ICU environment, declared that this kind of colonization was a potential risk to ICU patients, and predicted the possible threat that this OXA-232-CRKP clone would bring to public health.

### Long-term colonization of OXA-232-CRKP in the ICU.

During the 22-week surveillance, 8,656 samples were collected from various sites in the ICU, including 1,672 from patient colonization and 6,984 from environmental colonization. A total of 106 OXA-232-CRKPs were identified, with an average positive rate of 1.22% (Table 1; also see Table S1 in the supplemental material). The rate from the patient colonization was 3.59% (60/1,672 samples), over 5 times higher than that for the environment (0.66% [46/6,984 samples]), where rectal swabs and nasointestinal tubes presented the highest rates of 6.88% and 6.58%, respectively (Table 1). Additionally, it was widely colonized in the bedside areas of the environment as well, such as ventilator-related, bedside table, and micropump areas, with rates ranging from 0.18% to 1.52%. Samples from the ward areas were almost negative except for the sink-related site (1.42%) and switch button (0.58%) (Table 1). However, no strain was isolated from auxiliary areas, which might be because those devices were removable and the sampling frequencies were insufficient.

**TABLE 1:** Surveillance results on OXA-232-CRKP colonization in the ICU

Sample source^*a*^	Sampling frequency	No. of samples	No. (%) of OXA-232-CRKP strains
Patient-associated colonization sites		1,672	60 (3.59)
Rectal swab	Weekly	494	34 (6.88)
Nasointestinal tube	Weekly	76	5 (6.58)
Nasogastric tube	Weekly	282	9 (3.19)
Oral swab	Weekly	437	8 (1.83)
Tracheotomy tube	Weekly	253	4 (1.58)
Endotracheal intubation	Weekly	130	0 (0.00)
Environment-associated colonization sites		6,984	46 (0.66)
Bedside			
Ventilator related	Weekly	986	15 (1.52)
Bedside table	Weekly	586	6 (1.02)
Micropump	Weekly	499	4 (0.80)
ECG monitor	Weekly	585	4 (0.68)
Nebulizer	Weekly	494	3 (0.61)
Infusion stand	Weekly	587	2 (0.34)
Bed related	Weekly	1,164	3 (0.26)
Stethoscope	Weekly	543	1 (0.18)
Ward			
Sink related	Fortnightly	423	6 (1.42)
Switch button	Weekly	342	2 (0.58)
Crash trolley	Monthly	9	0 (0.00)
Dispensing trolley	Weekly	60	0 (0.00)
Locker	Weekly	290	0 (0.00)
Phone	Monthly	9	0 (0.00)
Computer related	Weekly	354	0 (0.00)
Door handle	Monthly	10	0 (0.00)
Auxiliary area			
Cleaning trolley related	Monthly	20	0 (0.00)
Ozone disinfector	Semiannually	1	0 (0.00)
Instrument cabinet	Monthly	13	0 (0.00)
Ultraviolet disinfection	Semiannually	1	0 (0.00)
Sputum elimination machine	Monthly	8	0 (0.00)
Total		8,656	106 (1.22)

a Ventilator related, ventilator and its shelf; bed related, bed rail and regulator; sink related, sink, faucet surface, inner surface of drain, inner wall of overflow, and tap water; computer related, computer keyboard and mouse, bar code machine, and scanner; cleaning trolley related, cleaning trolley cover, handle, and body and mop handle. ECG, electrocardiographic.

**FIG 1:**
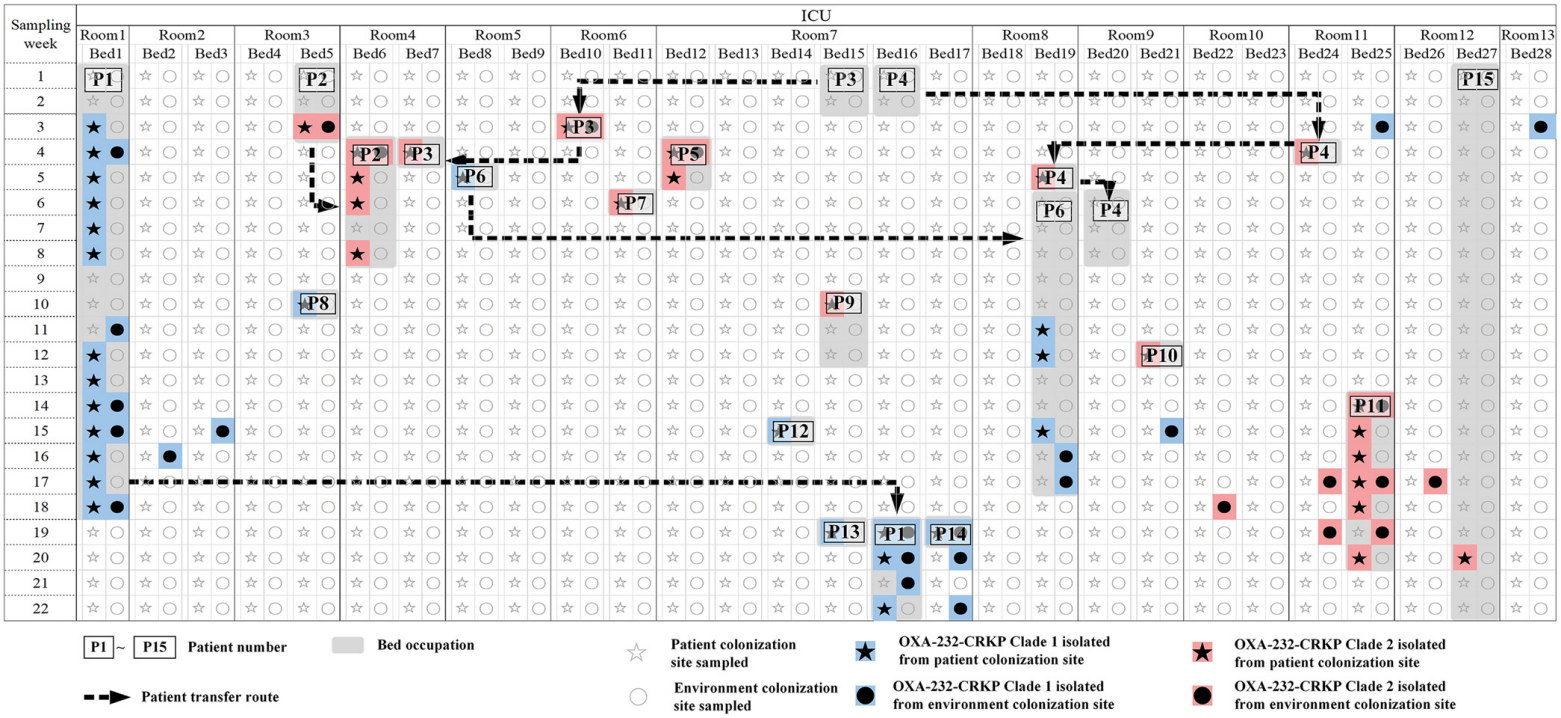
Schematic diagram of OXA-232-CRKPs and patients in the ICU during the 22-week surveillance. Stars and circles represent the sampling of colonization sites for patients and the environment, respectively. OXA-232-CRKP-positive sites are marked with blue or red shading, indicating clade 1 or clade 2 isolates, respectively, in the phylogenetic tree. OXA-232-CRKP strains at patient colonization sites were isolated from 15 patients in total, whose ICU occupation and transfer routes are marked with gray backgrounds and dashed arrows, respectively.

Regardless of where the OXA-232-CRKP was isolated, from the sites of patient colonization or the sites of the environment, most of the beds or rooms of the ICU examined had the isolation of OXA-232-CRKP consistently, except for bed 4 in room 4, bed 9 in room 5, bed 13 in room 7, bed 18 in room 8, and bed 23 in room 10 (Fig. 1, stars and circles in red or blue). Interestingly, it was observed that environmental colonization occurred along with related patient colonization. For instance, the positive detection of OXA-232-CRKP in the environment of bed 1 of room 1 lasted for almost 15 weeks in the same bed, whereas it appeared in bed 16 of room 7 after patient P1 was transferred. Similar patterns of strain transfer were also observed for patients P2, P3, P6, and P11, which indicated that such colonization status was related to patients and that patients might be the key source raising the spread of OXA-232-CRKP in the ICU. Meanwhile, there was a chance that the patient obtained the OXA-232-CRKP from environmental colonization. For instance, patient P15 in bed 27 of room 12 was not colonized by OXA-232-CRKP until the 20th week of the screening process, indicating that the OXA-232-CRKP from P15 might have been obtained from hospital environments.

### Colonization with OXA-232-CRKP in patients and confirmed infection.

As shown in Table 2, 15 patients (P1 to P15) who had OXA-232-CRKP isolated from the patient-associated colonization sites were traced successfully. These patients were mostly over 60 years of age (13 of 15 patients), suffered from complicated infections, solid tumors, or organ failure, and had undergone invasive procedures (13 of 15 patients). Unfortunately, 3 patients who underwent OXA-232-CRKP-related infection finally died, and 1 was only 40 years of age. Six patients (P1, P2, P3, P6, P8, and P11) were diagnosed with infections and produced another 11 OXA-232-CRKP isolates from their clinical specimens, including LF5, LF6, YHY1, LYD1, XGH1, XGH2, CYL1, CYL3, CYL4, ZLQ5, and ZLQ6 (Table 2; also see Table S1). Compared to the rest, patients P1, P2, P6, and P11 had OXA-232-CRKP colonization with more frequency. Notably, except for the patients who stayed in the ICU for only 1 week (P7, P8, P10, P12, P13, and P14), the average duration of colonization was 1.5 weeks in patients who were not infected by OXA-232-CRKP, while it was 9.2 weeks in patients with confirmed OXA-232-CRKP-associated infection (Fig. 1; also see Table S1).

**TABLE 2:** Information on patients who were confirmed to have patient-associated colonization or clinical infection by OXA-232-CRKP

Patient information^*a*^					Infection isolate information^*b*^	
Patient no.	Age (yr)/sex	Clinical diagnosis	Invasive procedure(s)	Outcome	Isolate identification no.	Specimen type (ward; date [yr/mo/day])
P1	92/M	Septicemia, septic shock, pneumonia	Endotracheal intubation, hemodialysis tube	Death	LF5 and LF6	Blood (ICU; 2020/5/27) and sputum (ICU; 2020/5/27)
P2	40/M	Cardiac arrest, pneumonia	Endotracheal intubation, hemodialysis tube	Death	YHY1	Sputum (ICU; 2019/8/20)
P3	83/F	Cerebral infarction, pneumonia	Tracheotomy, thoracocentesis	Discharge	LYD1	Sputum (ICU; 2019/8/9)
P4	65/M	Septic shock	Endotracheal intubation, abdominal puncture, hemodialysis tube	Discharge	ND	NA
P5	95/F	Acute heart failure, hydrothorax	Thoracocentesis	Discharge	ND	NA
P6	71/F	Septic shock, liver abscess, thoracic infection	Abdominal puncture, endotracheal intubation, hemodialysis tube	Discharge	XGH1 and XGH2	Purulent fluid (ICU; 2019/7/22) and hydrothorax (ICU; 2019/7/31)
P7	65/F	Type I respiratory failure	Endotracheal intubation	Discharge	ND	NA
P8	70/F	Systemic lupus erythematosus, septicemia	Endotracheal intubation, hemodialysis tube	Discharge	CYL1, CYL3 and CYL4	Blood (DR; 2019/5/8, 2019/7/19, and 2019/9/27)
P9	65/M	Intestinal obstruction, acute kidney injury	Abdominal puncture	Discharge	ND	NA
P10	36/M	Severe acute pancreatitis	Abdominal puncture, hemodialysis tube	Discharge	ND	NA
P11	74/M	Intestinal stromal tumor, abdominal infection, septicemia	Abdominal puncture, endotracheal intubation, hemodialysis tube	Death	ZLQ5 and ZLQ6	Ascites fluid (DID; 2019/10/20) and blood (ICU; 2020/1/11)
P12	88/M	Cardiac arrest, multisystem organ failure	—	Discharge	ND	NA
P13	69/M	Systemic lupus erythematosus, HBV hepatitis	Abdominal puncture, hemodialysis tube	Discharge	ND	NA
P14	68/M	Severe pneumonia (Chlamydia psittaci), type I respiratory failure	Endotracheal intubation	Discharge	ND	NA
P15	86/M	Motor neuron disease, respiratory failure	—	Discharge	ND	NA

a M, male; F, female; HBV, hepatitis B virus; —, no invasive procedure.

b ND, not detected (no OXA-232-CRKP); NA, not applicable; DR, Department of Respiratory; DID, Department of Infectious Disease.

According to previous reports, the infectious diseases caused by OXA-232-CRKP included pneumonia, urinary tract infections, and even septicemia (15, 23). Yang et al. reported an OXA-232-CRKP outbreak that was probably associated with endoscopic retrograde cholangiopancreatography procedures in a single institution in the United States, and 6 of 11 patients with OXA-232-CRKP infection died (24). Although the environment is a reservoir for nosocomial infection caused by multidrug-resistant pathogen (17), few reports have identified the effects of environmental colonization of OXA-232-CRKP on its nosocomial transmission. Here, we first investigated the colonization of OXA-232-CRKP in the environment and revealed that OXA-232-CRKP could colonize the environment sites continuously and be transmitted among different rooms, usually along with the transfer of patients. In addition, a longer duration of OXA-232-CRKP colonization could be a risk factor for secondary infection. The epidemic characteristics of OXA-232-CRKP in this program were further investigated through resistance profiling and WGS analysis.

### Resistance phenotypes of OXA-232-CRKP or transformants containing *bla*_OXA-232_.

All of the 117 OXA-232-CRKP isolates (106 from colonization samples and 11 from infection samples) presented identical profiles for antimicrobial agents; they were resistant to cefoxitin, ceftazidime, cefepime, piperacillin-tazobactam, cefoperazone-sulbactam, ertapenem, aztreonam, amikacin, and levofloxacin and presented variable rates of resistance to meropenem (88.89%) and imipenem (52.99%), whereas they were susceptible to ceftazidime-avibactam in all cases. The rates of resistance to colistin and tigecycline were 5.13% and 8.55%, respectively (see Table S2). The antibiotic resistance experiment suggested that the OXA-232-CRKP isolates presented a multidrug-resistant profile with different levels of resistance to carbapenems, as reported previously (23).

The contribution of *bla*_OXA-232_ and its plasmid to antibiotic resistance levels was verified through gene cloning experiments with different strains. Interestingly, the resistance phenotype caused by *bla*_OXA-232_ depended on its original plasmid and was related to the host species, exhibiting a higher level of carbapenem resistance in K. pneumoniae than in E. coli. In K. pneumoniae KP1107-151 (ST15 CRKP) cells, the pKP269-OXA232 plasmid increased the MICs of meropenem and ertapenem 8-fold and that of imipenem 2-fold. In E. coli DH5α, the MIC of ertapenem was increased 8-fold, while those of meropenem and imipenem were increased 2-fold by pKP269-OXA232 (see Table S3).

### Clonal spread of OXA-232-CRKP in the ICU.

High-risk clones (e.g., K. pneumoniae ST14, ST15, and ST17) have been associated with the global dispersion of OXA-232 (13, 25). In China, ST15 was the predominant multilocus sequence typing (MLST) type in nosocomial infections involving OXA-232-CRKP (9, 11, 15). Through WGS and single-nucleotide polymorphism (SNP) analysis, we revealed wide dissemination of 117 OXA-232-CRKP isolates due to clonal spread.

**FIG 2:**
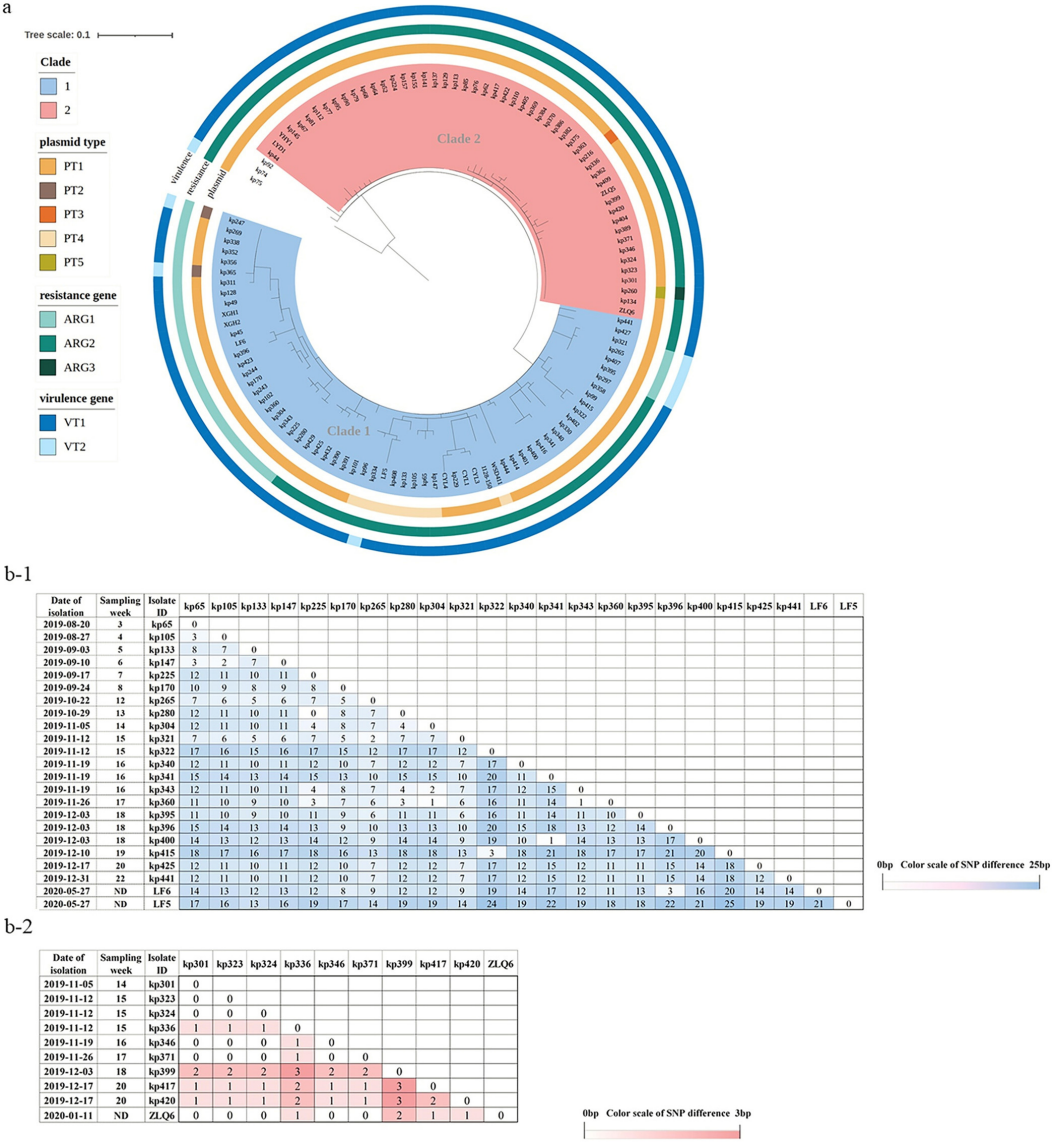
(a) Core genome phylogenetic tree of OXA-232-CRKP isolates generated by snippy and FastTree, including information about plasmid incompatibility types, resistance genes, and virulence genes. The core genome circle was divided into two predominant clades (clade 1 and clade 2), with differences of 0 to 26 SNPs and 0 to 10 SNPs, respectively. Three isolates that had a distant evolutionary relationship with other isolates were not classified into any clade according to FastTree analysis. Two strains, WSD411 and 1128-150, isolated in 2018 were phylogenetic references. The plasmid circle was separated into five PTs, PT1 to PT5. All of the 117 isolates contained four incompatibility types of plasmids, including IncFIB(pKPHS1), IncFII(K), ColRNAI, and ColE, while PT1 additionally had Col4401 and IncHl1B, PT2 had Col4401, PT3 had IncHI1B, PT4 had IncFIB (pQil), and PT5 had IncFII (pCRY). The ARG circle shows the ARGs contained by each isolate, ARG1 to ARG3. All isolates harbored the same ARGs, i.e., *oqxA6*, *oqxB20*, *bla*_CTX-M-15_, *bla*_SHV-106_, *fosA6*, *rmtF*, *arr-2*, *acc(6′)-lb*, *catB*, *qnrB1*, *dfrA14*, and *bla*_OXA-232_ which was defined as ARG1. ARG2 also had *bla*_TEM-1_, *sul2*, *aph(6)-ld*, and *aph(3′)-lb*, and ATG3 additionally contained *tet(A)*, *bla*_LAP2_, *qurS1*, and *catA2*. The virulence circle was divided into two groups; one (VT1) carried *rmpA2* and *iutAiucABCD* virulence genes, while the other (VT2) did not. (b) SNP matrix diagram of OXA-232-CRKP strains isolated from patient P1 (clade 1) (b-1) and patient P11 (clade 2) (b-2).

In our study, all OXA-232-CRKP isolates belonged to the same high-risk ST15 clone and serotype KL112. Furthermore, according to the SNP analysis, the OXA-232-CRKP ST15 clones were extremely close, with 0 to 45 SNP differences (Fig. 2a), while more than 2,000 SNPs between the OXA-232 and non-OXA-232-CRKP ST15 clones were detected (see Fig. S2), demonstrating that clonal spread instead of plasmid horizontal transmission was responsible for the wide dissemination of OXA-232-CRKP in our study. In addition, the isolates presented similar plasmid incompatibility types, antimicrobial resistance genes (ARGs), and virulence genes according to Illumina sequencing (Fig. 2a). For the plasmid incompatibility type, there were five plasmid type (PT) compositions (PT1 to PT5), and all 117 isolates contained four incompatibility types of plasmids, including IncFIB(pKPHS1), IncFII(K), ColRNAI, and ColE. Then, ARGs could be divided into three groups (ARG1 to ARG3). ARG1 existed in 28 isolates that were clustered in clade 1, including ARGs conferring resistance to β-lactams (*bla*_OXA-232_, *bla*_CTX-M-15_, and *bla*_SHV-106_) and aminoglycosides (*rmtF1*) (other ARGs are listed in Fig. 2a). Compared to ARG1, ARG2 additionally carried *bla*_TEM-1_, *aph(6)-ld*, *aph(3′)-lb*, and *sul2*, which existed in 88 isolates. All isolates in clade 2 contained ARGs that belonged to ARG2, except for one OXA-232-CRKP strain that was isolated from a nasogastric tube and harbored *tet(A)*, *bla*_LAP2_, *qurS1*, and *catA2* in addition, defined as ARG3. Lastly, 109 OXA-232-CRKP isolates (93.16% [109/117 isolates]) carried *rmpA2* and *iutAiucABCD*, which was classified as virulence type 1 (VT1), and isolates that lacked the virulence determinants were VT2 (Fig. 2a).

Above all, the 117 OXA-232-CRKP strains were transmitted among environment sites, patient colonization sites, and even patient infection sites through ST15 clonal spread. One reason for the wide clonal spread was that the OXA-232 plasmid brought no fitness cost to its host in Mueller-Hinton (MH) culture, resulting in the notable transmission ability of OXA-232-CRKP (see Fig. S3). Another important reason could be that all of the strains in our study carried the disinfectant resistance gene *cepA*, which encodes a multidrug efflux pump; *cepA* was reported to induce resistance to chlorhexidine, a widely used disinfectant with broad-spectrum bactericidal activity, and to facilitate the wide dispersal of pathogen through the environment transmission medium (26).

**FIG 3:**
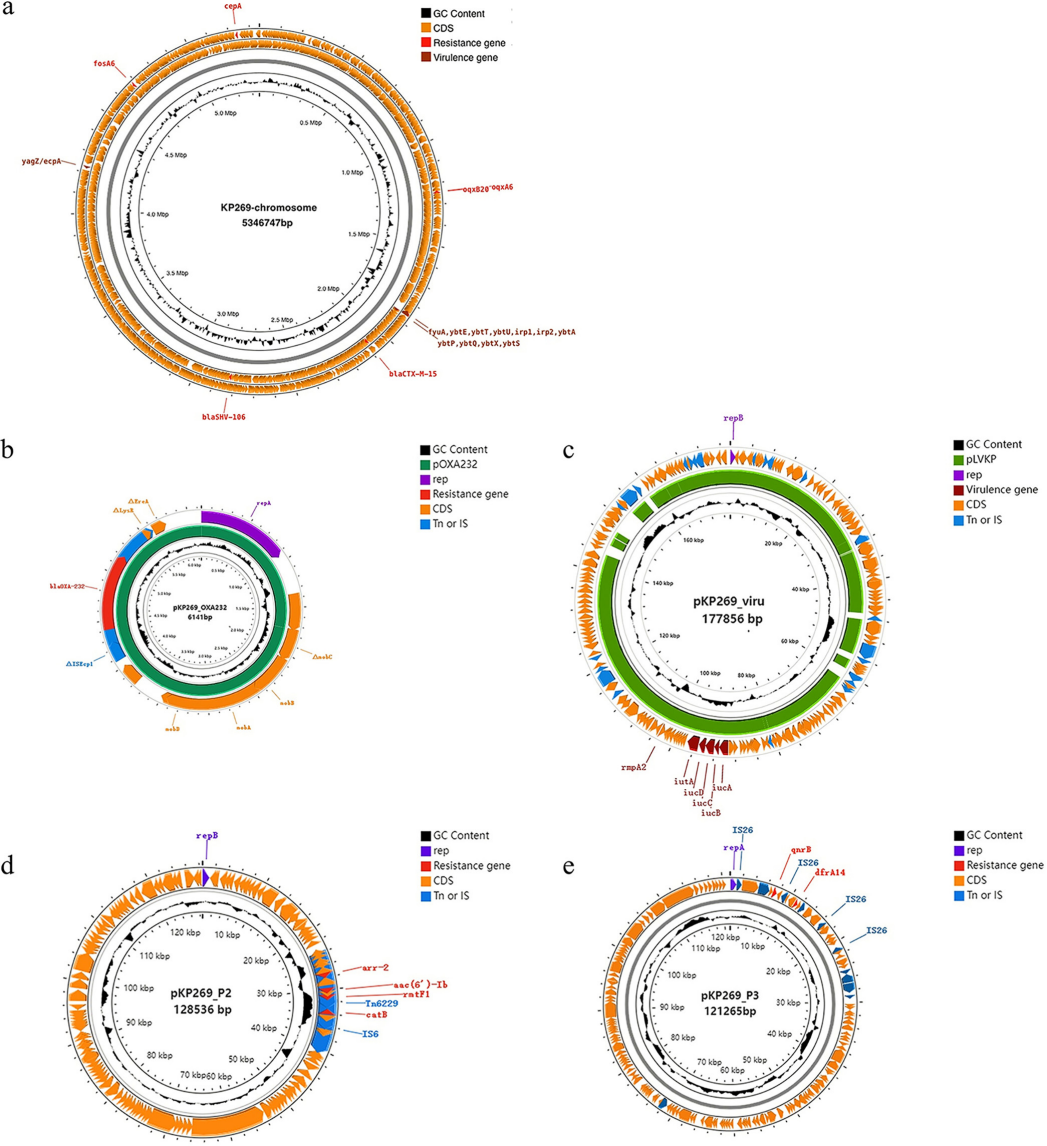
Genomic structure of the chromosome and plasmids harboring resistance genes or virulence genes in OXA-232-CRKP KP269. (a) The chromosome carried virulence genes, ARGs, and a disinfectant resistance gene (*cepA*). CDS, coding DNA sequences. (b) The ColE plasmid (pKP269_OXA232) contained only one resistance gene, *bla*_OXA-232_, possessing a backbone similar to that of pOXA-232 (GenBank accession number JX423831), the first reported *bla*_OXA-232_ plasmid in the world (100% coverage and 99.98% identity). (c) The IncHI1B-type plasmid (pKP269_viru) carrying virulence determinants (*rmpA2* and *iutAiucABCD*) presents high levels of homology with the classic virulence plasmid pLVPK (GenBank accession number AY378100), with 92% coverage and 99.56% identity. (d and e) IncFIB(pKPHS1)-type (d) and IncFII(K)-type (e) resistance gene-carrying plasmids.

To determine the specific location of ARGs and virulence genes in detail, KP269 was selected for long-read sequencing. One chromosome and six plasmids of KP269 were identified (Fig. 3; two plasmids with no resistance or virulence genes are not presented). The chromosome, which was 5,346,747 bp in length, carried five ARGs, including *oqxA6*, *oqxB20*, *bla*_CTX-M-15_, *bla*_SHV-106_, and *fosA6*. In addition, there were virulence genes, including *ybtA/E/P/Q/S/T/U/X*, *irp1/2*, *fyuA*, and *yagZ/ecpA*, and the disinfectant resistance gene *cepA* on the chromosome (Fig. 3a). The *bla*_OXA-232_ gene was located on a ColE-type plasmid, pKP269_OXA232, with a size of 6,141 bp, which is the same as the pOXA232 reported previously (GenBank accession number JX423831) in sequence, with 100% coverage and 99.98% identity (12) (Fig. 3b). Notably, pKP269_viru is a typical virulence plasmid containing determinants of *rmpA2* and *iutAiucABCD*. It was 177.8 kb in length, belonged to the IncHI1B group, and showed similarity to pLVPK (GenBank accession number AY378100), with 92% coverage and 99.56% identity (Fig. 3c). pKP269_P2, an IncFIB-type plasmid, carried ARGs, including *rmtF1*, *arr-2*, *acc(6′)-Ib*, and *cat(B)*, which were located in Tn*6229* (Fig. 3d). Furthermore, pKP269-3 contained *qnrB* and *dfrA14* but, compared to the plasmid in strain WSD411, pKP269_P3 lacked *bla*_CTX-M-15_, *bla*_TEM-1_, *aph(6)-Id*, *aph(3′)-Ib*, and *sul2*, which were due to insertion sequence (IS) IS*26* (Fig. 3e).

The OXA-232-CRKP clone has the potential to obtain carbapenemase and virulence determinants. On the one hand, the coexistence of *bla*_OXA-232_ and *bla*_NDM-1 or -5_ in K. pneumoniae has been reported, with meropenem and imipenem MICs of >64 mg/L (27). In our study, one non-OXA-232-ST15 CRKP, which produced KPC-2, belonged to the same cluster as the OXA-232-CRKP (see Fig. S2), suggesting that the OXA-232-CRKP ST15 clone had the potential to obtain *bla*_KPC-2_. Palavecino et al. reported the discovery of KPC-2- and OXA-232-coproducing K. pneumoniae in the United States (28). On the other hand, most OXA-232-CRKP ST15 clones (93.16% [109/117 isolates]) in our study carried *rmpA2* (hypermucoidy) and *iutAiucABCD* (aerobactin siderophore) virulence clusters, which usually exist in hypervirulent clones, such as ST23. RmpA2 can promote the production of capsules, which play an important role in immune evasion from host cells and help hypervirulent K. pneumoniae (hvKP) invade the bloodstream (29). As an important virulence factor, aerobactin notably enhanced the growth of hvKP in human ascites (30). Similarly, Shu et al. isolated 10 OXA-232-CRKP ST15 clones carrying pLVPK-like virulence plasmids from elderly patients in China (15). The fact that the high-risk OXA-232-CRKP ST15 clone has the potential to acquire virulence plasmids and other carbapenemases is a threat to public health.

### Microevolution and transmission of OXA-232-CRKP in the ICU.

According to core genome SNP analysis, the differences in SNPs among the OXA-232-CRKP strains were between 0 and 45 SNPs, and the strains could be clearly divided into two clades on the SNP phylogenetic tree, suggesting microevolution progress during clonal spread (Fig. 2a).

The SNP difference within clade 1 was 0 to 26 SNPs, and that within clade 2 was 0 to 10 SNPs. The two reference isolates (WSD411 and 1107-151) from 2018 were clustered in clade 1, indicating that those isolates of clade 1 could be traced back to 1 year earlier. Figure 2a shows that isolates in clade 1 presented more diversity, with two main kinds of ARGs and VTs. Isolates from P1 (clade 1) and P11 (clade 2), who were continuously colonized and then suffered from septicemia associated with OXA-232-CRKP, were selected for the evolution analysis (Fig. 2b). With time, the SNP difference of strains isolated from the same patients gradually increased. Moreover, the average SNP distance was 10.67 and 0.99 in colonization isolates from P1 and P11 over 20 and 7 weeks, respectively, indicating that isolates from P1 in clade 1 evolved faster. Shu et al. reported ST15 clone dissemination among elderly patients over 3 months, differing by only 0 to 15 SNPs (15). It was speculated that the reason for more SNP differences in our study could be that OXA-232-CRKP evolved to produce more mutation sites to adapt to the ICU environment with the extension of colonization time.

Strains belonging to the same clade were considered to have a transmission relationship, and the distribution of OXA-232-CRKP is displayed in Fig. 1. OXA-232-CRKP was observed to be transmitted among the patient and environment colonization sites. The transmission events could be divided into two modes, including transmission of OXA-232-CRKP within a bed unit associated with the same patient and transmission between different bed units in the same room or even across rooms. According to the surveillance, ventilator- and sink-related sites and bedside tables were the most common environmental colonization sites for OXA-232-CRKP. Notably, the ventilator and bedside tables were frequently operated by nurses, as a result of which it was suggested that reminders should be placed at these places and medical care workers should be informed to perform hand disinfection with alcohol after exposure to these sites, as well as touching a patient (31). In addition, contaminated sinks were identified as reservoirs of multidrug-resistant Pseudomonas aeruginosa and *Enterobacteriaceae* (16, 17). Sometimes the use of sinks is not strictly classified; for instance, sinks could be used for the disposal of bodily fluid wastes in addition to handwashing (32). Thus, to prevent transmission through the sinks, it is necessary to prohibit functions of the sink except for handwashing. Moreover, considering the emergence of the disinfectant resistance gene in OXA-232-CRKP clones, continuous use of the same kind of disinfectant should be avoided in cases of resistance (33).

A limitation of our study is that the mechanism of different carbapenem resistance levels was not clear. In OXA-232-CRKP, the reported mechanism of high-level carbapenem resistance included *ompk* mutations and a high copy number of the OXA-232 plasmid (11, 34). However, all OXA-232-CRKP isolates in our study shared the same carbapenem resistance-related *ompk36* (A217S) and *ompk37* (I70M and I128M) mutations (35), and there was no significant relationship between plasmid copy number and carbapenem susceptibility in our study (see Fig. S1). The mechanism of high-level carbapenem resistance needs to be further investigated.

### Conclusion.

In conclusion, we reported nosocomial dissemination of OXA-232-CRKP dominated by ST15 clonal spread among the environment and patients in the ICU. The OXA-232-CRKP clone presented a multidrug resistance profile, carrying a disinfectant resistance gene and virulence clusters. Our results revealed the long-term colonization of OXA-232-CRKP ST15 in the ICU environment and declared that this kind of colonization was a potential risk to ICU patients. The rapid transmission represents a worrisome step in the rise of high-risk OXA-232-CRKP ST15 clones in hospitals. Thus, reasonable usage of disinfectants could be critical to avoid the emergence of disinfectant resistance genes and to prevent widespread OXA-232-CRKP dissemination in the ICU.

## MATERIALS AND METHODS

### Program design.

To surveil the colonization of OXA-232-CRKP in the ICU, a program was scheduled in SRRSH (Zhejiang Province, China) from 1 August to 31 December 2019. The ICU involved has a total of 28 beds (bed 1 to bed 28), which are divided into 13 separate rooms (room 1 to room 13), 10 of which have 2 beds, 2 of which have 1 bed, and 1 of which has 6 beds (Fig. 1). Samples were sourced from patient or environment colonization sites with different frequencies, as described in a previous report (36) (Table 1). The initial screening of CRKP was performed by using Simmon’s citrate agar plates (Haibo, Qingdao, China) with 1% inositol and 2 mg/L ertapenem, followed by matrix-assisted laser desorption ionization–time of flight mass spectrometry (MALDI-TOF MS) (Vitek mass spectrometer; bioMérieux). Then, PCR and sequencing were used for the confirmation of the *bla*_OXA-232_ gene with the primers OXA-232-F (5′-ATGCGTGTATTAGCCTTATCGGC-3′) and OXA-232-R (5′-CTAGGGAATAATTTTCTCCTGTTTGA-3′).

To investigate the relationship between colonization of OXA-232-CRKP and patient infection, we traced the medical histories of the patients who were positive for OXA-232-CRKP from the colonization sites. Those patients who had infection symptoms in their medical histories and had OXA-232-CRKP in their clinical specimens were defined as having OXA-232-CRKP-related infections. Here, OXA-232-CRKP isolation from patients was first completed according to a standard culture method, and then the isolates were screened for *bla*_OXA-232_ by PCR and sequencing with the same primers, OXA-232-F and OXA-232-R.

The medical history was analyzed based on information provided by the Hospital Information System (HIS) under approval granted by the ethics committee of SRRSH (approval number 20201217-33).

### Antimicrobial susceptibility testing.

Antimicrobial susceptibility testing (AST) with cefoxitin, ceftazidime, cefepime, piperacillin-tazobactam, cefoperazone-sulbactam, meropenem, imipenem, ertapenem, aztreonam, amikacin, levofloxacin, ceftazidime-avibactam, tigecycline, and colistin were performed according to the Clinical and Laboratory Standards Institute (CLSI) guideline M07 (37), and MIC breakpoints from the CLSI guideline M100 were used for the interpretation of susceptibility except for tigecycline, which was determined according to the FDA 2019 guideline (resistance at ≥8 μg/mL) (https://www.fda.gov/drugs/development-resources/tigecycline-injection-products) (38).

### Resistance phenotype of the *bla*_OXA-232_ gene.

The TA DNA cloning method was applied by using the OXA-232-CRKP isolate KP269 as the cloning subject according to standard procedures with minor modifications. First, genomic DNA of KP269 was extracted with the QIAamp DNA minikit (Qiagen, USA) and then was used as a template for PCR amplification. A fragment of the *bla*_OXA-232_ gene and its predicted promoter were amplified with the primers TA-OXA-232-F (5′-TAAGAATATCATCAATAAAATTGAGTGTTG-3′) and TA-OXA-232-R (5′-CTAGGGAATAATTTTCTCCTGTTTGA-3′). After being confirmed by sequencing, the fragment was cloned into the pCR2.1 vector using the T4 DNA ligase kit (Invitrogen, Shanghai, China) according to the manufacturer’s instructions, and the ligation products were transformed into competent E. coli DH5α cells using the heat shock method. MH plates with 50 mg/L kanamycin were used for the selection of transformants of E. coli DH5α containing the pCR2.1-OXA232 plasmid. Colonies growing on the selection plates were analyzed for *bla*_OXA-232_ gene by PCR. The pCR2.1 vector was introduced into E. coli DH5α as a blank control. AST was then applied to detect the MICs of different antibiotics for the transformants [E. coli DH5α(pCR2.1-OXA232) and E. coli DH5α(pCR2.1)].

KP269 was prepared for resistance confirmation of the *bla*_OXA-232_ gene at the plasmid level. KP269 was first extracted from the whole plasmid using a Qiagen plasmid extraction kit and then transformed into E. coli DH5α and K. pneumoniae KP1107-151 at the same time (39). KP1107-151 is an ST15 CRKP without all known carbapenemases, including OXA-232. The follow-up selection procedures for transformed E. coli DH5α and KP1107-151 were almost the same as above except for the addition of 0.015 mg/L or 8 mg/L ertapenem, respectively. Colonies that were positive for *bla*_OXA-232_ by PCR, named DH5α(pKP269-OXA232) or KP1107-151(pKP269-OXA232), were further set up for AST.

### WGS and analysis.

Genomic DNA was extracted using a QIAamp DNA minikit (Qiagen) and subjected to Illumina paired-end sequencing (Illumina Inc., San Diego, CA). One OXA-232-CRKP isolate, KP269, was selected for further long-read Nanopore sequencing (Oxford Nanopore Technologies, Oxford, UK). Sequence reads generated by Illumina sequencing were assembled by Shovill 0.9.0 (https://github.com/tseemann/shovill), and long-read Nanopore reads were assembled using Unicycler v0.4.8 (40). ARGs were screened using ABRicate with the NCBI database, and PTs were identified using the PlasmidFinder database (https://github.com/tseemann/abricate). The BIGSdb-Kp and Kaptive databases were used for the detection of virulence genes and K-antigen capsular typing, respectively (https://bigsdb.pasteur.fr/klebsiella/). MLST was performed with the Center for Genomic Epidemiology online tool (http://cge.cbs.dtu.dk/services/MLST).

SNPs among strains were determined using Snippy v4.4.5 (https://github.com/tseemann/snippy) by using KP269 as a reference sequence. FastTree v2.2.10 was then used for building of the SNP phylogenetic tree. Additionally, the genomic sequence data for two OXA-232-CRKP strains (strain 1128-150, unpublished data; strain WSD411, GenBank accession number CP045686; both isolated in 2018 from SRRSH) were included in this study for phylogenetic reference. The WGS data for 28 OXA-232-negative ST15 CRKP (non-OXA232-ST15) strains (isolated from SRRSH; X. H. Han, unpublished data) were included in clonality analysis of ST15.

The copy number of the *bla*_OXA-232_ plasmid (ColE-OXA-232 plasmid) was calculated according to a previous report (41). The sequence contigs generated by Illumina sequencing were mapped to the genomic sequence of KP269. The average coverage of contigs mapping to the ColE-OXA-232 plasmid was taken as the coverage of the ColE-OXA-232 plasmid, and the average coverage of the contigs in which the seven housekeeping genes (*gapA*, *infB*, *mdh*, *pgi*, *phoE*, *rpoB*, and *tonB*) were located was taken as the coverage of the chromosome. The relative copy number was obtained by comparing the contig coverage of the ColE-OXA-232 plasmid with that of the chromosome.

### Growth rate measurement.

To evaluate the fitness cost to K. pneumoniae KP1107-151 (pKP269-OXA232) after obtaining the resistant plasmid, a growth experiment comparing it with the parent strain in a Bioscreen C MBR instrument (Oy Growth Curves Ab Ltd., Finland) was completed (42). Triple independent cultures were incubated overnight at 37°C and diluted to 1:100 in MH broth. Optical density at 600 nm (OD_600_) values for the replicates were then measured every 10 min for 20 h. The growth rates were estimated based on the OD_600_ values using an R script. Differences in the mean growth rates were assessed by Student's *t* test using GraphPad Prism v7. *P* values of <0.05 were considered significant.

### Data availability.

The draft genomes of non-OXA232-ST15 strains were under BioProject ID: PRJNA807050.
