# Supplementary material for: Epidemiological Characteristics of OXA-232-Producing Carbapenem-Resistant Klebsiella pneumoniae Strains Isolated during Nosocomial Clonal Spread Associated with Environmental Colonization
Source: Microbiol Spectr. 2022 Jun 22;10(4):e02572-21. doi: 10.1128/spectrum.02572-21 (PMC9430510; doi:10.1128/spectrum.02572-21)
Supplement: Supplemental file 1 — Supplemental material. Download spectrum.02572-21-s0001.pdf, PDF file, 0.4 MB [file spectrum.02572-21-s0001.pdf]

Supplemental table 1. Basic information of OXA-232-CRKP isolates in this study

No.	Isolate ID	Sampling week	Date of isolation	Department	Bed in ICU	Room in ICU	Patient number	Group	Sample source	Clade	PT	ARG	VT
1	kp65	3	2019-08-20	ICU	B1	R1	P1	PC	Rectal swab	Clade 1	PT4	ARG2	VT1
2	kp105	4	2019-08-27	ICU	B1	R1	P1	PC	Rectal swab	Clade 1	PT4	ARG2	VT1
3	kp133	5	2019-09-03	ICU	B1	R1	P1	PC	Rectal swab	Clade 1	PT4	ARG2	VT1
4	kp147	6	2019-09-10	ICU	B1	R1	P1	PC	Rectal swab	Clade 1	PT4	ARG2	VT1
5	kp225	7	2019-09-17	ICU	B1	R1	P1	PC	Rectal swab	Clade 1	PT1	ARG1	VT1
6	kp170	8	2019-09-24	ICU	B1	R1	P1	PC	Rectal swab	Clade 1	PT1	ARG1	VT1
7	kp265	12	2019-10-22	ICU	B1	R1	P1	PC	Rectal swab	Clade 1	PT1	ARG1	VT2
8	kp280	13	2019-10-29	ICU	B1	R1	P1	PC	Rectal swab	Clade 1	PT1	ARG1	VT1
9	kp304	14	2019-11-05	ICU	B1	R1	P1	PC	Rectal swab	Clade 1	PT1	ARG1	VT1
10	kp321	15	2019-11-12	ICU	B1	R1	P1	PC	Rectal swab	Clade 1	PT1	ARG1	VT2
11	kp322	15	2019-11-12	ICU	B1	R1	P1	PC	Nasogastric tube	Clade 1	PT1	ARG2	VT1
12	kp340	16	2019-11-19	ICU	B1	R1	P1	PC	Tracheotomy tube	Clade 1	PT1	ARG2	VT1
13	kp341	16	2019-11-19	ICU	B1	R1	P1	PC	Nasointestinal tube	Clade 1	PT1	ARG2	VT1
14	kp343	16	2019-11-19	ICU	B1	R1	P1	PC	Rectal swab	Clade 1	PT1	ARG1	VT1
15	kp360	17	2019-11-26	ICU	B1	R1	P1	PC	Rectal swab	Clade 1	PT1	ARG1	VT1
16	kp395	18	2019-12-03	ICU	B1	R1	P1	PC	Rectal swab	Clade 1	PT1	ARG1	VT2

17	kp396	18	2019-12-03	ICU	B1	R1	P1	PC	Tracheotomy tube	Clade 1	PT1	ARG1	VT1
18	kp400	18	2019-12-03	ICU	B1	R1	P1	PC	Nasogastric tube	Clade 1	PT1	ARG2	VT1
19	kp52	3	2019-08-20	ICU	B5	R3	P2	PC	Oral swab	Clade 2	PT1	ARG2	VT1
20	kp64	3	2019-08-20	ICU	B5	R3	P2	PC	Rectal swab	Clade 2	PT1	ARG2	VT1
21	kp229	10	2019-10-08	ICU	B5	R3	P8	PC	Rectal swab	Clade 1	PT1	ARG2	VT1
22	kp113	4	2019-08-27	ICU	B6	R4	P2	PC	Nasogastric tube	Clade 2	PT1	ARG2	VT1
23	kp79	4	2019-08-27	ICU	B6	R4	P2	PC	Oral swab	Clade 2	PT1	ARG2	VT1
24	kp81	4	2019-08-27	ICU	B6	R4	P2	PC	Rectal swab	Clade 2	PT1	ARG2	VT1
25	kp129	5	2019-09-03	ICU	B6	R4	P2	PC	Rectal swab	Clade 2	PT1	ARG2	VT1
26	kp145	5	2019-09-03	ICU	B6	R4	P2	PC	Oral swab	Clade 2	PT1	ARG2	VT1
27	kp157	6	2019-09-10	ICU	B6	R4	P2	PC	Rectal swab	Clade 2	PT1	ARG2	VT1
28	kp224	8	2019-09-24	ICU	B6	R4	P2	PC	Rectal swab	Clade 2	PT1	ARG2	VT1
29	kp75	4	2019-08-27	ICU	B7	R4	P3	PC	Rectal swab	-	PT1	ARG2	VT2
30	kp85	4	2019-08-27	ICU	B7	R4	P3	PC	Oral swab	Clade 2	PT1	ARG2	VT1
31	kp128	5	2019-09-03	ICU	B8	R5	P6	PC	Nasointestinal tube	Clade 1	PT1	ARG1	VT1
32	kp62	3	2019-08-20	ICU	B10	R6	P3	PC	Rectal swab	Clade 2	PT1	ARG2	VT1
33	kp155	6	2019-09-10	ICU	B11	R6	P7	PC	Rectal swab	Clade 2	PT1	ARG2	VT1
34	kp112	4	2019-08-27	ICU	B12	R7	P5	PC	Nasogastric tube	Clade 2	PT1	ARG2	VT1
35	kp77	4	2019-08-27	ICU	B12	R7	P5	PC	Oral swab	Clade 2	PT1	ARG2	VT1

36	kp137	5	2019-09-03	ICU	B12	R7	P5	PC	Oral swab	Clade 2	PT1	ARG2	VT1
37	kp141	5	2019-09-03	ICU	B12	R7	P5	PC	Nasogastric tube	Clade 2	PT1	ARG2	VT1
38	kp352	15	2019-11-12	ICU	B14	R7	P12	PC	Rectal swab	Clade 1	PT1	ARG1	VT1
39	kp414	19	2019-12-10	ICU	B15	R7	P13	PC	Rectal swab	Clade 1	PT1	ARG2	VT1
40	kp216	10	2019-10-08	ICU	B15	R7	P9	PC	Rectal swab	Clade 2	PT3	ARG2	VT1
41	kp415	19	2019-12-10	ICU	B16	R7	P1	PC	Tracheotomy tube	Clade 1	PT1	ARG2	VT1
42	kp425	20	2019-12-17	ICU	B16	R7	P1	PC	Tracheotomy tube	Clade 1	PT1	ARG2	VT1
43	kp441	22	2019-12-31	ICU	B16	R7	P1	PC	Rectal swab	Clade 1	PT1	ARG2	VT1
44	kp416	19	2019-12-10	ICU	B17	R7	P14	PC	Rectal swab	Clade 1	PT1	ARG2	VT1
45	kp134	5	2019-09-03	ICU	B19	R8	P4	PC	Nasointestinal tube	Clade 2	PT1	ARG2	VT1
46	kp247	11	2019-10-15	ICU	B19	R8	P6	PC	Nasogastric tube	Clade 1	PT2	ARG1	VT2
47	kp269	12	2019-10-22	ICU	B19	R8	P6	PC	Nasointestinal tube	Clade 1	PT1	ARG1	VT1
48	kp311	15	2019-11-12	ICU	B19	R8	P6	PC	Rectal swab	Clade 1	PT1	ARG1	VT1
49	kp260	12	2019-10-22	ICU	B21	R9	P10	PC	Nasogastric tube	Clade 2	PT5	ARG3	VT1
50	kp76	4	2019-08-27	ICU	B24	R11	P4	PC	Nasogastric tube	Clade 2	PT1	ARG2	VT1
51	kp301	14	2019-11-05	ICU	B25	R11	P11	PC	Rectal swab	Clade 2	PT1	ARG2	VT1
52	kp323	15	2019-11-12	ICU	B25	R11	P11	PC	Nasogastric tube	Clade 2	PT1	ARG2	VT1
53	kp324	15	2019-11-12	ICU	B25	R11	P11	PC	Oral swab	Clade 2	PT1	ARG2	VT1
54	kp336	15	2019-11-12	ICU	B25	R11	P11	PC	Rectal swab	Clade 2	PT1	ARG2	VT1

55	kp346	16	2019-11-19	ICU	B25	R11	P11	PC	Rectal swab	Clade 2	PT1	ARG2	VT1
56	kp371	17	2019-11-26	ICU	B25	R11	P11	PC	Rectal swab	Clade 2	PT1	ARG2	VT1
57	kp399	18	2019-12-03	ICU	B25	R11	P11	PC	Nasointestinal tube	Clade 2	PT1	ARG2	VT1
58	kp417	20	2019-12-17	ICU	B25	R11	P11	PC	Oral swab	Clade 2	PT1	ARG2	VT1
59	kp420	20	2019-12-17	ICU	B25	R11	P11	PC	Rectal swab	Clade 2	PT1	ARG2	VT1
60	kp422	20	2019-12-17	ICU	B27	R12	P15	PC	Rectal swab	Clade 2	PT1	ARG2	VT1
61	kp101	4	2019-08-27	ICU	B1	R1	ND	EC	Ventilator	Clade 1	PT1	ARG2	VT1
62	kp102	4	2019-08-27	ICU	B1	R1	ND	EC	Inner surface of drain	Clade 1	PT1	ARG2	VT1
63	kp96	4	2019-08-27	ICU	B1	R1	ND	EC	Bed regulator	Clade 1	PT4	ARG2	VT1
64	kp99	4	2019-08-27	ICU	B1	R1	ND	EC	Nebuliser	Clade 1	PT1	ARG2	VT1
65	kp243	11	2019-10-15	ICU	B1	R1	ND	EC	Bed rail	Clade 1	PT1	ARG1	VT1
66	kp244	11	2019-10-15	ICU	B1	R1	ND	EC	Inner wall of overflow	Clade 1	PT1	ARG1	VT1
67	kp297	14	2019-11-05	ICU	B1	R1	ND	EC	Bed regulator	Clade 1	PT1	ARG2	VT1
68	kp334	15	2019-11-12	ICU	B1	R1	ND	EC	Switch button	Clade 1	PT4	ARG2	VT2
69	kp390	18	2019-12-03	ICU	B1	R1	ND	EC	Ventilator	Clade 1	PT1	ARG2	VT1
70	kp391	18	2019-12-03	ICU	B1	R1	ND	EC	Micropump	Clade 1	PT1	ARG2	VT1
71	kp358	16	2019-11-19	ICU	B2	R2	ND	EC	Ventilator shelf	Clade 1	PT1	ARG2	VT1

72	kp330	15	2019-11-12	ICU	B3	R2	ND	EC	Bedside Table	Clade 1	PT1	ARG2	VT1
73	kp67	3	2019-08-20	ICU	B5	R3	ND	EC	Ventilator	Clade 2	PT1	ARG2	VT1
74	kp68	3	2019-08-20	ICU	B5	R3	ND	EC	Bedside Table	Clade 2	PT1	ARG2	VT1
75	kp95	4	2019-08-27	ICU	B6	R4	ND	EC	Ventilator	Clade 2	PT1	ARG2	VT1
76	kp44	3	2019-08-20	ICU	B10	R6	ND	EC	Stethoscope	Clade 2	PT1	ARG2	VT1
77	kp74	3	2019-08-20	ICU	B10	R6	ND	EC	ECG monitor	-	PT1	ARG2	VT1
78	kp92	4	2019-08-27	ICU	B10	R6	ND	EC	Bedside Table	-	PT1	ARG2	VT1
79	kp90	4	2019-08-27	ICU	B12	R7	ND	EC	Micropump	Clade 2	PT1	ARG2	VT1
80	kp402	19	2019-12-10	ICU	B16	R7	ND	EC	ECG monitor	Clade 1	PT1	ARG2	VT1
81	kp407	19	2019-12-10	ICU	B16	R7	ND	EC	Ventilator	Clade 1	PT1	ARG1	VT2
82	kp408	19	2019-12-10	ICU	B16	R7	ND	EC	Bedside Table	Clade 1	PT4	ARG2	VT1
83	kp423	20	2019-12-17	ICU	B16	R7	ND	EC	Ventilator shelf	Clade 1	PT1	ARG1	VT1
84	kp429	21	2019-12-24	ICU	B16	R7	ND	EC	Ventilator shelf	Clade 1	PT1	ARG1	VT1
85	kp432	21	2019-12-24	ICU	B16	R7	ND	EC	Ventilator	Clade 1	PT1	ARG2	VT1
86	kp401	19	2019-12-10	ICU	B17	R7	ND	EC	Micropump	Clade 1	PT1	ARG2	VT1
87	kp427	20	2019-12-17	ICU	B17	R7	ND	EC	Ventilator	Clade 1	PT1	ARG2	VT1
88	kp444	22	2019-12-31	ICU	B17	R7	ND	EC	ECG monitor	Clade 1	PT1	ARG2	VT1
89	kp356	16	2019-11-19	ICU	B19	R8	ND	EC	Ventilator	Clade 1	PT4	ARG1	VT1
90	kp365	17	2019-11-26	ICU	B19	R8	ND	EC	Bedside Table	Clade 1	PT2	ARG1	VT2

91	kp338	15	2019-11-12	ICU	B21	R9	ND	EC	Ventilator	Clade 1	PT1	ARG1	VT1
92	kp389	18	2019-12-03	ICU	B22	R10	ND	EC	Nebuliser	Clade 2	PT1	ARG2	VT1
93	kp382	17	2019-11-26	ICU	B24	R11	ND	EC	ECG monitor	Clade 2	PT1	ARG2	VT1
94	kp405	19	2019-12-10	ICU	B24	R11	ND	EC	Switch button	Clade 2	PT1	ARG2	VT1
95	kp310	14	2019-11-05	ICU	B25	R11	ND	EC	Ventilator	Clade 2	PT1	ARG2	VT1
96	kp362	17	2019-11-26	ICU	B25	R11	ND	EC	Ventilator	Clade 2	PT1	ARG2	VT1
97	kp363	17	2019-11-26	ICU	B25	R11	ND	EC	Ventilator shelf	Clade 2	PT1	ARG2	VT1
98	kp369	17	2019-11-26	ICU	B25	R11	ND	EC	Infusion stand	Clade 2	PT1	ARG2	VT1
99	kp370	17	2019-11-26	ICU	B25	R11	ND	EC	Sink	Clade 2	PT1	ARG2	VT1
100	kp375	17	2019-11-26	ICU	B25	R11	ND	EC	Micropump	Clade 2	PT1	ARG2	VT1
101	kp386	17	2019-11-26	ICU	B25	R11	ND	EC	Inner surface of drain	Clade 2	PT1	ARG2	VT1
102	kp404	19	2019-12-10	ICU	B25	R11	ND	EC	Inner wall of overflow	Clade 2	PT1	ARG2	VT1
103	kp409	19	2019-12-10	ICU	B25	R11	ND	EC	Inner surface of drain	Clade 2	PT1	ARG2	VT1
104	kp49	3	2019-08-20	ICU	B25	R11	ND	EC	Nebuliser	Clade 1	PT1	ARG1	VT1
105	kp384	17	2019-11-26	ICU	B26	R12	ND	EC	Infusion stand	Clade 2	PT1	ARG2	VT1
106	kp45	3	2019-08-20	ICU	B28	R13	ND	EC	Bedside Table	Clade 1	PT1	ARG1	VT1

107	LF5	ND	2020-05-27	ICU	B8	R5	P1	PI	Blood	Clade 1	PT4	ARG2	VT1
108	LF6	ND	2020-05-27	ICU	B8	R5	P1	PI	Sputum	Clade 1	PT1	ARG1	VT1
109	YHY1	ND	2019-08-20	ICU	B4	R3	P2	PI	Sputum	Clade 2	PT1	ARG2	VT1
110	LYD1	ND	2019-08-09	ICU	B15	R7	P3	PI	Sputum	Clade 2	PT1	ARG2	VT1
111	XGH1	ND	2019-07-22	ICU	B9	R5	P6	PI	Purulent fluid	Clade 1	PT1	ARG1	VT1
112	XGH2	ND	2019-07-31	ICU	B16	R7	P6	PI	Hydrothorax	Clade 1	PT1	ARG1	VT1
113	CYL1	ND	2019-05-08	DR	ND	ND	P8	PI	Blood	Clade 1	PT1	ARG2	VT1
114	CYL3	ND	2019-07-19	DR	ND	ND	P8	PI	Blood	Clade 1	PT1	ARG2	VT1
115	CYL4	ND	2019-09-27	DR	ND	ND	P8	PI	Blood	Clade 1	PT1	ARG2	VT1
116	ZLQ5	ND	2019-10-20	DID	ND	ND	P11	PI	Ascite fluid	Clade 2	PT1	ARG2	VT1
117	ZLQ6	ND	2020-01-11	ICU	B25	R11	P11	PI	Blood	Clade 2	PT1	ARG2	VT1

ND, not detected. Department: ICU, Intensive Care Unit. DR, Department of Respiratory. DID, Department of Infectious Disease. Group: PC, Patient-associated colonization; EC, Environment-associated colonization; PI, Patient confirmed infection. PT, Plasmid incompatibility type. ARG, antimicrobial resistance gene type. VT, virulence type.

Supplemental table 2. Antimicrobial susceptibility result of 117 OXA-232-CRKP in our study

Antimicrobial agent	Rate (%)			MIC parameters（mg/L）		
	S	I	R	MIC Range	MIC50	MIC90
FOX	0	0	100	32->64	>64	>64
CAZ	0	0	100	16->32	>32	>32
FEP	0	0	100	>32	>32	>32
TZP	0	0	100	256->256	>256	>256
CPS	0	0	100	128->128	>128	>128
MEM	3.42	7.69	88.89	1->128	4	32
IPM	11.11	35.90	52.99	0.5->128	4	64
ETP	0	0	100	4->128	64	>128
ATM	0	0	100	32->32	>32	>32
AMK	0	0	100	>128	>128	>128
LEV	0	0	100	>4	>4	>4
CZA	100	0	0	0.125-1	0.5	1
TGC	82.05	9.40	8.55	0.06-8	2	4
CST	/	94.87	5.13	0.06->64	0.125	0.5

FOX, cefoxitin; CAZ, ceftazidime; FEP, cefepime; TZP, piperacillin-tazobactam; CPS, cefoperazone-sulbactam; MEM, meropenem; IPM, imipenem; ETP, ertapenem; ATM, aztreonam; AMK, amikacin; LEV, levofloxacin; CZA, ceftazidime-avibactam; TGC, tigecycline; CST, colistin. S, susceptible; I, intermediate; R, resistant. /, No susceptible MIC breakpoint for *K. pneumoniae* to colistin according to CLSI guideline.

Supplemental table 3. Antimicrobial susceptibility result of transformers obtaining *bla*$_{OXA-232}$ gene or plasmid (mg/L)

Antimicrobial agent	*K. pneumoniae*			*E. coli*			
	KP269	KP1107-151	KP1107-151 (pKP269-OXA232)	DH5α	DH5α (pKP269-OXA232)	DH5α (pCR2.1)	DH5α (pCR2.1-OXA232)
FOX	>64	64	64	4	4	4	4
CAZ	>64	>64	>64	0.25	0.25	0.25	0.25
FEP	>32	>32	>32	0.125	0.125	0.125	0.125
TZP	>256	**128**	**>512**	**1**	**32**	**1**	**16**
CPS	>128	128	>128	**0.06**	**8**	**0.06**	**4**
MEM	8	**1**	**8**	0.03	0.06	0.03	0.06
IMP	4	0.5	1	0.125	0.25	0.125	0.125
ETP	64	**4**	**64**	**0.0075**	**0.06**	0.0075	0.015
ATM	>32	>32	>32	0.03	0.03	0.03	0.03
AMK	>128	16	16	0.5	0.5	2	2
LEV	>4	0.5	0.5	0.03	0.03	0.03	0.03

FOX, cefoxitin; CAZ, ceftazidime; FEP, cefepime; TZP, piperacillin-tazobactam; CPS, cefoperazone-sulbactam; MEM, meropenem; IPM, imipenem; ETP, ertapenem; ATM, aztreonam; AMK, amikacin; LEV, levofloxacin. Number in bold means MIC change over two-fold dilution.

Supplemental Figure legends:

Figure S1. Relationship between ColE-OXA-232 plasmid copy number and carbapenem susceptibility.

Figure S2. Phylogenetic tree of CRKP isolates of the ST15 clone. The isolates included all OXA-232-CRKP in our study and 28 *bla*$_{\text{OXA-232}}$ negative ST15 CRKP strains (non-OXA-232-ST15). All OXA-232-CRKPs were clustered into Clade a. One non-OXA-232-ST15 strain, kp115, was divided into the Clade a, while other non-OXA-232-ST15 strains had more than 2000 SNPs compared with OXA-232-CRKP, and were clustered into Clade b.

Figure S3. Comparison of fitness cost between KP1107-151 and KP1107-151 (pKP269-OXA232) in the aspects of the growth rates (a) and the growth curves (b).

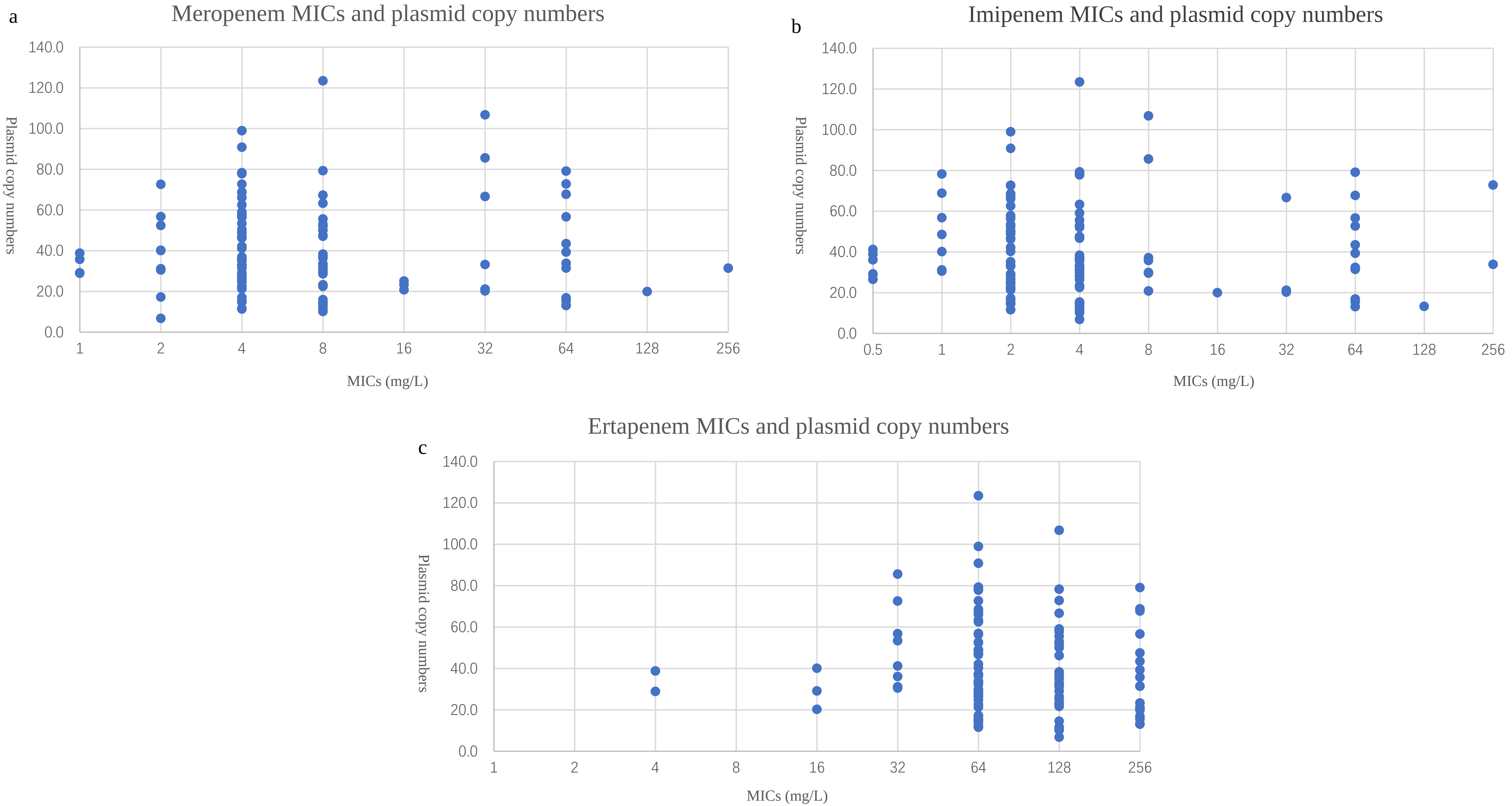
a
Meropenem MICs and plasmid copy numbers
Plasmid copy numbers
MICs (mg/L)
0.0
20.0
40.0
60.0
80.0
100.0
120.0
140.0
1
2
4
8
16
32
64
128
256
b
Imipenem MICs and plasmid copy numbers
Plasmid copy numbers
MICs (mg/L)
0.5
1
2
4
8
16
32
64
128
256
c
Ertapenem MICs and plasmid copy numbers
Plasmid copy numbers
MICs (mg/L)

Figure S1

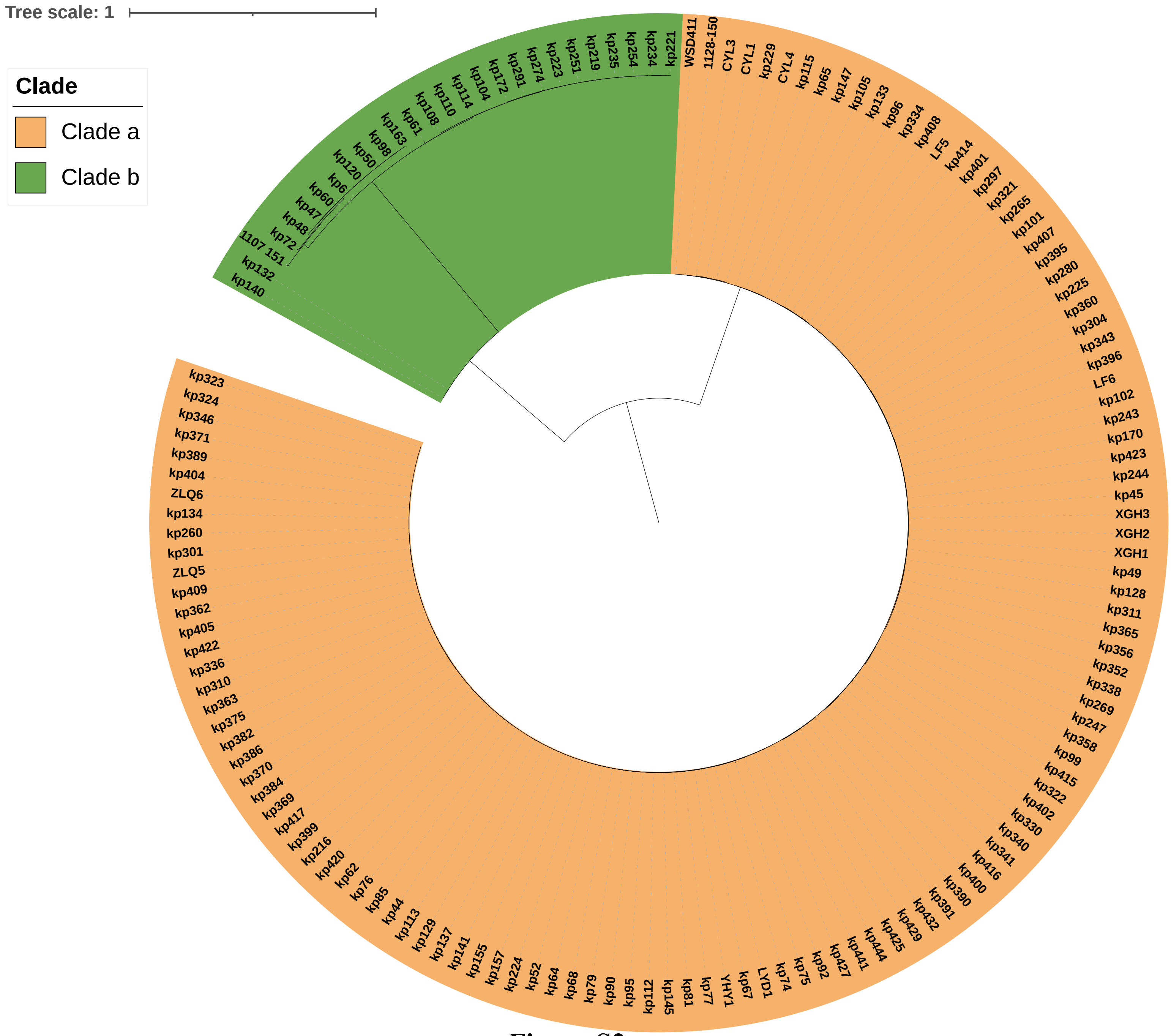
Tree scale: 1
Clade
Clade a
Clade b
kp221
kp234
kp254
kp235
kp219
kp251
kp223
kp274
kp291
kp172
kp104
kp114
kp110
kp108
kp61
kp163
kp98
kp50
kp120
kp6
kp60
kp47
kp48
kp72
1107 151
kp132
kp140
WSD411
1128-150
CYL3
CYL1
kp229
CYL4
kp115
kp65
kp147
kp105
kp133
kp96
kp334
kp408
LF5
kp414
kp401
kp297
kp321
kp265
kp101
kp407
kp395
kp280
kp225
kp360
kp304
kp343
kp396
LF6
kp102
kp243
kp170
kp423
kp244
kp45
XGH3
XGH2
XGH1
kp49
kp128
kp311
kp365
kp356
kp352
kp338
kp269
kp247
kp358
kp99
kp415
kp322
kp402
kp330
kp340
kp341
kp416
kp400
kp390
kp391
kp432
kp429
kp425
kp444
kp441
kp427
kp92
kp75
kp74
LYD1
kp67
YHY1
kp77
kp81
kp145
kp112
kp95
kp90
kp79
kp68
kp64
kp52
kp224
kp157
kp155
kp141
kp137
kp129
kp113
kp44
kp85
kp76
kp62
kp420
kp216
kp399
kp417
kp369
kp384
kp370
kp386
kp382
kp375
kp363
kp310
kp336
kp422
kp405
kp362
kp409
ZLQ5
kp301
kp260
kp134
ZLQ6
kp404
kp389
kp371
kp346
kp324
kp323

Figure S2

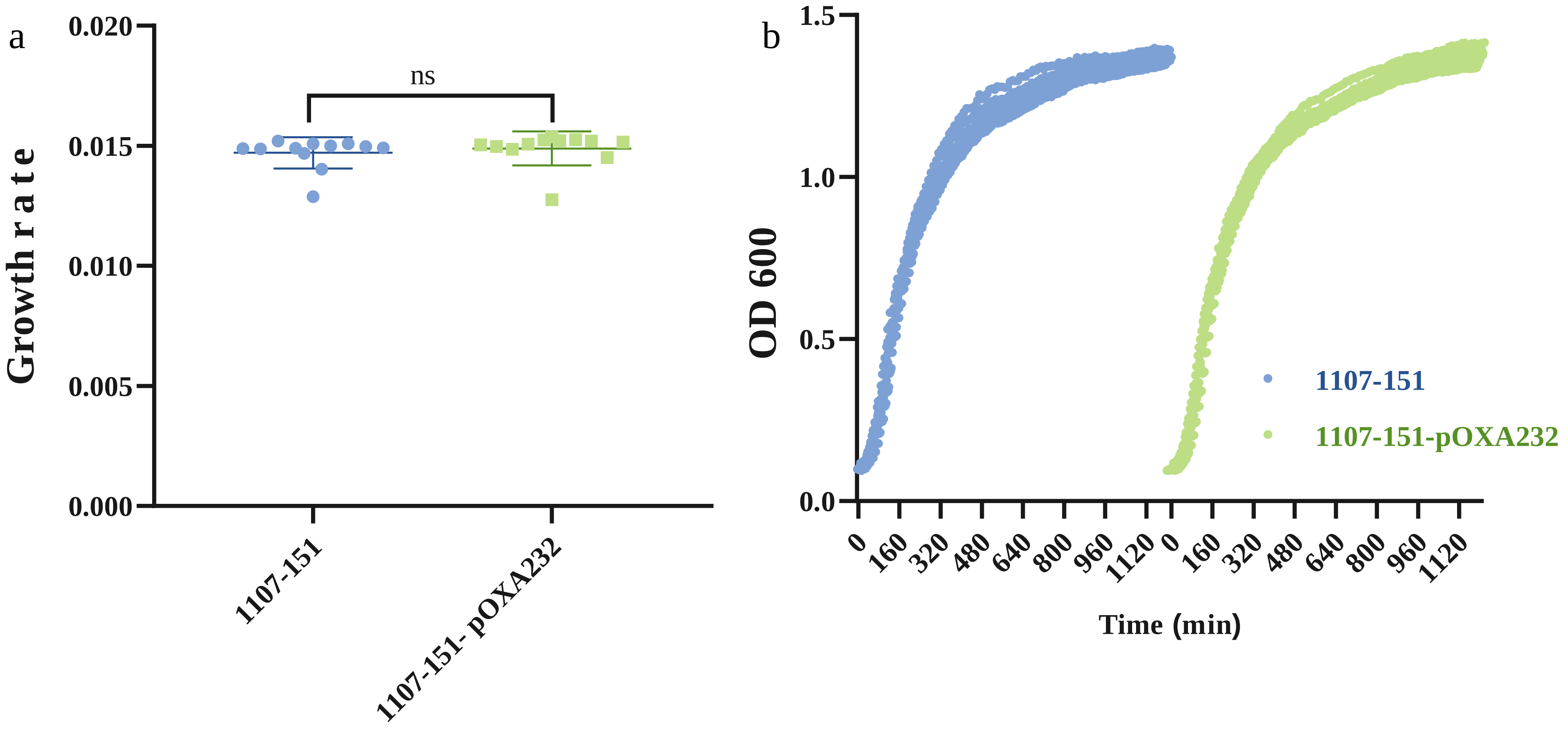
a
Growth rate
0.020
0.015
0.010
0.005
0.000
ns
1107-151
1107-151- pOXA232
b
OD 600
1.5
1.0
0.5
0.0
0
160
320
480
640
800
960
1120
Time (min)
1107-151
1107-151-pOXA232

Figure S3
